# Genome-Wide Characterization of GRAS Family and Their Potential Roles in Cold Tolerance of Cucumber (*Cucumis sativus* L.)

**DOI:** 10.3390/ijms21113857

**Published:** 2020-05-29

**Authors:** Xiaohong Lu, Wenqian Liu, Chenggang Xiang, Xiaojun Li, Qing Wang, Tao Wang, Zixi Liu, Jiali Zhang, Lihong Gao, Wenna Zhang

**Affiliations:** 1 Beijing Key Laboratory of Growth and Developmental Regulation for Protected Vegetable Crops, China Agricultural University, Beijing 100193, China; luxh0317@163.com (X.L.); lwq08@cau.edu.cn (W.L.); 15933020677@163.com (X.L.); bolukesi@163.com (Q.W.); 18222736601@163.com (T.W.); xideyouxiang99@163.com (Z.L.); zhangjialisc@163.com (J.Z.); lhgao@cau.edu.cn (L.G.); 2 College of Life Science and Technology, HongHe University, Mengzi 661100, China; z0501080535@126.com

**Keywords:** genome-wide, GRAS, *Cucumis sativus*, expression profile, phylogeny, cold tolerance

## Abstract

Cucumber (*Cucumis sativus* L.) is one of the most important cucurbit vegetables but is often subjected to stress during cultivation. *GRAS* (*gibberellic acid insensitive, repressor of GAI, and scarecrow*) genes encode a family of transcriptional factors that regulate plant growth and development. In the model plant *Arabidopsis thaliana*, GRAS family genes function in formation of axillary meristem and root radial structure, phytohormone (gibberellin) signal transduction, light signal transduction and abiotic/biological stress. In this study, a gene family was comprehensively analyzed from the aspects of evolutionary tree, gene structure, chromosome location, evolutionary and expression pattern by means of bioinformatics; 37 GRAS gene family members have been screened from cucumber. We reconstructed an evolutionary tree based on multiple sequence alignment of the typical GRAS domain and conserved motif sequences with those of other species (*A. thaliana* and *Solanum lycopersicum*). Cucumber GRAS family was divided into 10 groups according to the classification of *Arabidopsis* and tomato genes. We conclude that tandem and segmental duplication have played important roles in the expansion and evolution of the cucumber GRAS (*CsaGRAS*) family. Expression patterns of *CsaGRAS* genes in different tissues and under cold treatment, combined with gene ontology annotation and interaction network analysis, revealed potentially different functions for *CsaGRAS* genes in response to cold tolerance, with members of the SHR, SCR and DELLA subfamilies likely playing important roles. In conclusion, this study provides valuable information and candidate genes for improving cucumber tolerance to cold stress.

## 1. Introduction

Transcription factors, also known as trans-acting elements, can activate or inhibit gene expression by binding to 5′-flank cis-elements of target genes, thereby regulating plant growth and development [1]. The GRAS family of plant-specific transcription factors exists widely in plants [2], including *Arabidopsis* [3], grapevine (*Vitis vinifera*) [4], Chinese cabbage (*Brassica rapa* ssp. *pekinensis*) [5], tomato (*Solanum lycopersicum)* [6], lotus (*Nelumbo nucifera*) [7] and mustard (*Brassica juncea*) [8].

Named for three functional proteins: GAI (gibberellic acid insensitive) [9], RGA (repressor of GA1-3 mutant) [10], and SCR (scarecrow) [11], proteins of the GRAS family generally comprise 400–700 amino acids [12], although this range is expanded in some species, e.g., 318–792 amino acids [8] in mustard. Typical transcription factors are composed of a DNA-binding domain, transcriptional regulatory (activation or repression) domain, oligomerization site and nuclear localization signals [13]. GRAS proteins contain a variable N-terminal and a highly conserved C-terminal region. The conserved C-terminal of typical GRAS has five different sequence motifs: leucine heptad repeat I (LHRI, LRI), VHIID motif, leucine heptad repeat II (LHRII, LRII), PFYRE motif and SAW motif [14]. The LRI, VHIID, LRII motifs or the LRI-VHIID-LRII pattern may be the DNA-binding or protein-binding regions of GRAS proteins, which function in protein interactions. Additionally, the PFYRE and SAW motifs may be related to the structural integrity of GRAS proteins [15]. However, not all N-terminal regions of GRAS are intrinsically disordered. For example, DELLA proteins contain a conserved DELLA motif in the N-terminal region, which are described to be involved in molecular recognition [16].

GRAS family genes function in various biological processes in plants, such as gibberellin signal transduction [9,10,17], axillary meristem formation [18], root radial pattern formation [11,19], male gametogenesis [13], photosensitive pigment A signal transduction [20] and nodulation signal transduction [21], and biotic and abiotic stress resistance [22]. GRAS family genes are large and their structures are diverse; research has focused not only on mining GRAS families in different plants, but also on exploring the functions of their members and classifying them according to their sequence, structure and phylogenetic relationships. The GRAS family in model plants (*Oryza sativa* L. and *Arabidopsis*) can be divided into eight groups: LISCL, PAT1, SCL3, DELLA, SCR, SHR, LS and HAM [3]. Identification of additional GRAS protein functions in different plants has allowed further division of the GRAS family, with at least nine groups in lotus [7] and mustard [8], 10 groups in tomato [23] and at least 13 groups in tea trees (*Camellia sinensis*) [24]. Studies have shown that GRAS proteins are involved in different metabolic processes in different subfamilies. For example, LS (LAS) subfamily genes are involved in the processes of inflorescence number [12], flowering time [25], flowering induction [26] and lateral bud growth [27,28]. In addition, HAM subfamily genes are highly expressed in flowers [12]. The moving transcriptional regulator, SHR, will form the specific heterodimer SHR–SCR, and the SHR–SCR complex is involved in controlling plant organ development [13,29,30]. N-terminal DELLA motif subfamily proteins, such as AtGAI, OsSLR1 and ZmD8, play a negative regulatory role in the gibberellic acid (GA) signaling pathway. After binding to GA, GID1(GA-insensitive dwarf 1) recognizes and interacts with DELLA proteins, inducing their degradation through the 26S proteasome, and thereby, relieving inhibition of the GA signal [15,31]; the level of bioactive gibberellin can be inhibited by *CBF1* that involved in the CBF/DREB1 (the C-repeat binding factor/dehydration-responsive element binding factor 1) pathway; and the CBF/DREB1 pathway is an important cold-signal reaction pathway [32,33], thereby inhibiting plant growth and enhancing cold tolerance [34]. PAT1, SCL21 and SCL13 belong to the PAT1 branch of the GRAS family. PAT1 and SCL21 are positive regulators of phytochrome A (PhyA) signal transduction, and SCL13 is independently involved in the phytochrome B (PhyB) pathway [35,36]; phyB, a thermosensor of ambient temperature, can mediate plant responses to environmental temperature [37,38].

Cucumber (*Cucumis sativus* L.) is widely cultivated in temperate and tropical regions, where plants are generally sensitive to cold. Cold stress causes damage related to metabolic imbalance, because enzymes in metabolic pathways and various cellular processes are differentially inhibited by cold [39]. Given the importance of GRAS transcription factors in regulating metabolism and cellular processes, and the high amino acid identity among members of each GRAS subfamily that suggests similar or related functions [3], we aimed to characterize the GRAS family from cucumber. We identified 37 *CsaGRAS* genes through genome-wide analysis of the cucumber GRAS family with reference to studies in *Arabidopsis* and tomato. We focused on gene expression patterns under cold stress to elucidate potential functions in response to cold. Our characterization of cold-responsive *CsaGRAS* genes provides candidate genes and new perspective for understanding the molecular evolution and biological functions of the GRAS family.

## 2. Results

### 2.1. Genome-Wide Identification of GRAS Family Members in Cucumber

Through screening the cucumber genome database, we identified 37 genes encoding GRAS protein domains. Gene ID, amino acid length, protein molecular weight, theoretical isoelectric point (PI), grand average of hydropathicity (GRAVY) and amino acid composition associated with each gene are shown in Table S1. The estimated length of cucumber GRAS proteins was 388–857 amino acids, with molecular weight of 44.83–92.74 kDa, PI of 4.76–7.63 and average aliphatic index of 82.62. The GRAVY values of all GRAS proteins were negative (average −0.30), indicating that these proteins are hydrophilic.

### 2.2. Evolutionary Relationships and Classification of GRAS Family Genes

**Figure 1:**
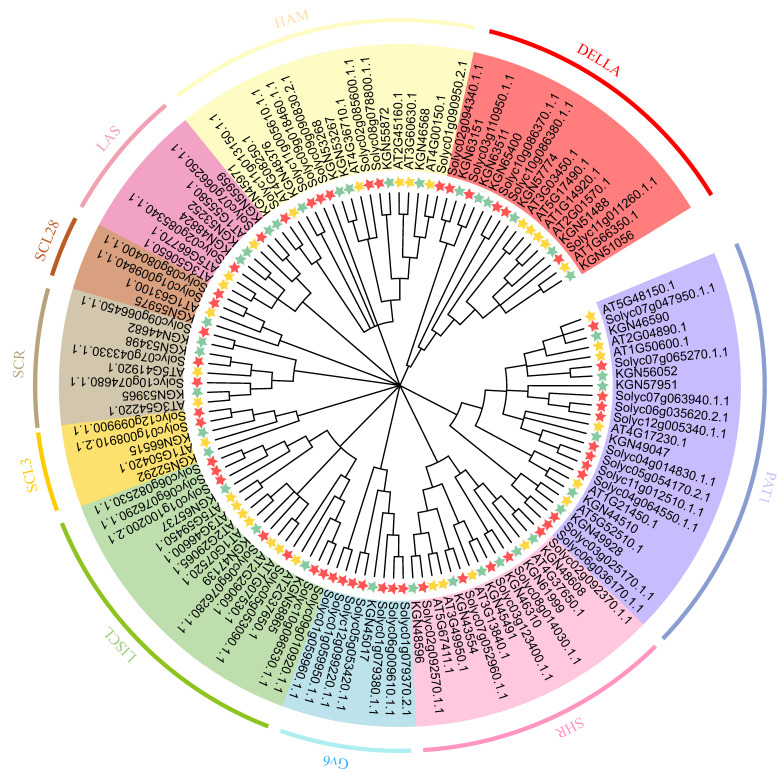
Phylogenetic tree of *CsaGRAS* genes in Cucumber, Arabidopsis and Tomato. The phylogenetic tree was constructed by the Neighbor-joining method, with 1000 bootstrap repeats. 10 classifies are represented by different colors, and the name of each subfamily is also marked in the corresponding position. The *GRAS* genes of the three species are represented by different colors stars: green for cucumber, yellow for Arabidopsis and red for tomato.

To understand the phylogenetic relationships and classification of *CsaGRAS* genes, we reconstructed a phylogenetic tree on the basis of multiple sequence alignment of 122 protein sequences, including 37 from cucumber, 34 from *Arabidopsis* and 51 from tomato. According to the classification of *Arabidopsis* and tomato GRAS proteins, all GRAS proteins were divided into 10 clusters: LISC, PAT1, SCL3, DELLA, SCR, SHR, LAS, SCL28, Gv6 and HAM (Figure 1; Supplementary Table S2) [32,38]. The *GRAS* genes of the three species were irregularly distributed across the 10 subfamilies. Consistent with previous studies [8,23], PAT1 was one of the largest subfamilies, accounting for 18.85% of the total *GRAS* genes (122); SCL4 and SCL28 were the smallest subfamilies (Table 1). Cucumber had more GRAS transcription factors belonging to the PTA1, SHR, HAM and DELLA subfamilies than to the other subfamilies, consistent with other species [7,8,23], and the distribution proportion of *GRAS* genes among the 10 subfamilies in cucumber was more similar to that in *Arabidopsis* than to that in tomato, except for the GV6 subfamily that comprise seven tomato, one cucumber and no *Arabidopsis GRAS* genes. Homologous genes were often clustered, indicating that cucumber *GRAS* genes have a closer evolutionary relationship to those of *Arabidopsis* than to those of tomato, and therefore, *Arabidopsis* may provide a better reference. Members of the same subfamily had similar gene structure and function, allowing us to hypothesize the function of the uncharacterized genes. For example, AT3G03450.1 and AT1G66350.1 belong to the DELLA subfamily and regulate warmth acclimation in cold-acclimated *Arabidopsis* [40]. We hypothesize that cucumber DELLA subfamily genes (such as *Csa5G421370*) play a crucial part in cold tolerance in cucumber.

### 2.3. Structural Analysis of CsaGRAS Genes

**Figure 2:**
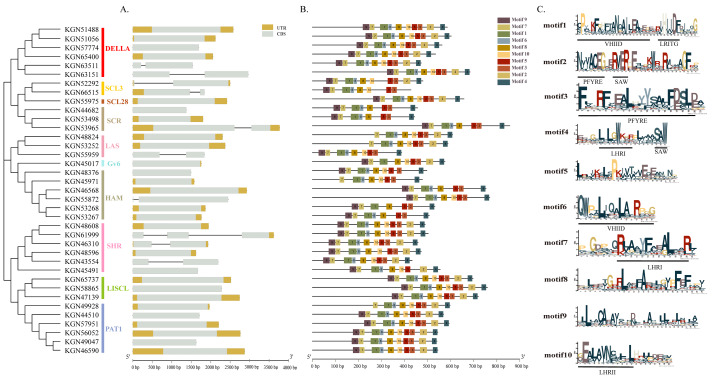
Structural analysis of *CsaGRAS* genes in cucumber. (**A**) The exon-intron structure of *GRAS* gene. (**B**) The distribution of motif in GRAS proteins. (**C**) The amino acid composition of each motif.

Structural analysis of *GRAS* genes allowed us to comprehensively understand the conserved characteristics of cucumber GRAS proteins and analyze differences in their evolution. The proportion of cucumber *GRAS* genes without introns was 54.05%, while the other genes contained only 1–2 introns. There was some subfamily specificity: members of the PAT1 and LISCL subfamilies did not contain introns; and the gene containing two introns was in the SHR subfamily, possibly attributed to an increase or decrease in the number of introns during evolution (Figure 2A). Motif analysis indicated that all GRAS proteins contained > 7 motifs, of which 51.35% contained 10 motifs (Figure 2B,C; the amino acid sequences of the 10 motifs are listed in Supplementary Table S3). In addition, proteins located on the same branch of the phylogenetic tree had a similar number and arrangement of motifs. For example, members of both DELLA and LISCL subfamilies contained 10 motifs, while those of the SCR subfamily contained eight motifs (lacking motif2 and motif10). However, the distribution of motifs in the HAM subfamily was quite different; it might be due to this subfamily not being sectionalized in sufficient detail. For example, the evolutionary relationships of KGN46568(Csa_6G109640) and KGN55872(Csa_3G020600) were closer to AT2G45160.1 (SCL27) and AT3G60630.1 (SCL22) of *Arabidopsis* (Figure 1). Motifs were more likely to be distributed at the C-terminal than the N-terminal. We identified different parts of five GRAS domains (LHRI, LHRII, VHIID, PFYRE and SAW described previously [3]) in the 10 motifs (Figure 2C). Comparing the length of each gene, intron-exon structure and motifs, we observed that the genes of each subfamily had similar characteristics, indicating that the majority of *CsaGRAS* genes were conserved among the three species.

### 2.4. Distribution and Duplication of GRAS Genes in Cucumber

**Figure 3:**
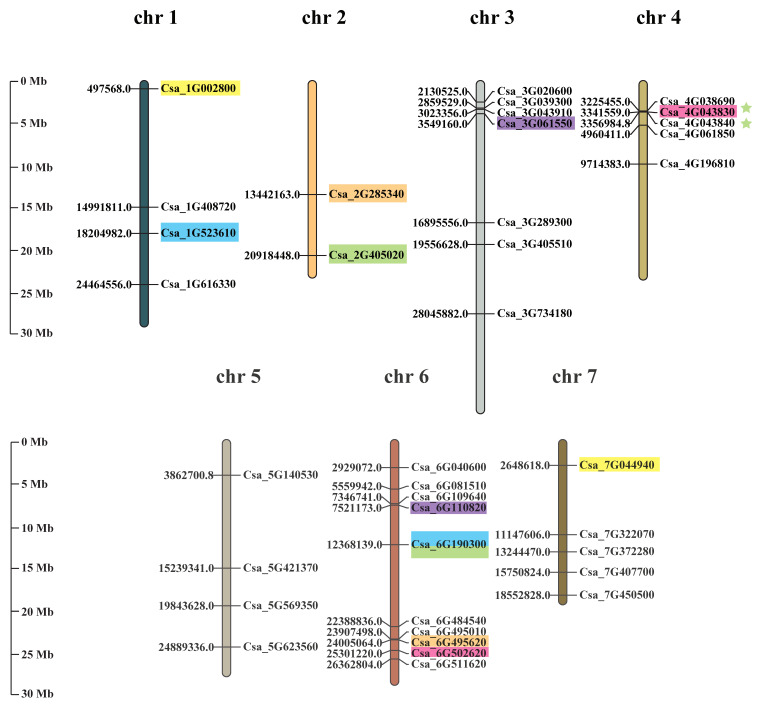
Chromosome mapping and duplication of *CsaGRAS* genes in cucumber. The number on the left side of the chromosome is the starting position, and on the right is the gene ID; segmental duplication gene pairs are marked with different color blocks and tandem duplication gene pairs are marked with green stars.

**Figure 4:**
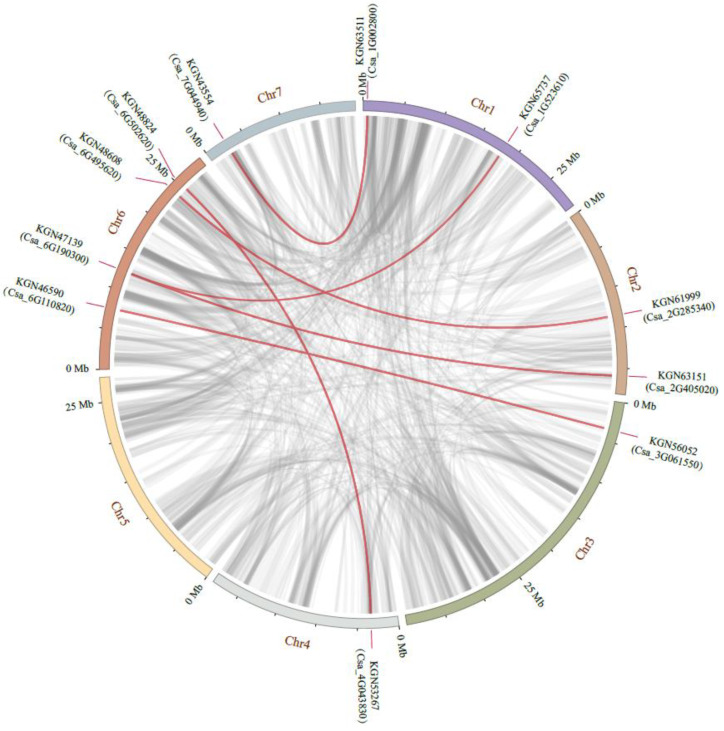
Chromosome localization of CsaGRAS duplicated genes in cucumber. The red lines represent the segmentally duplicated genes and the black bands represent the collinear block.

The *CsaGRAS* genes were unevenly distributed on seven chromosomes, with none located on unanchored contigs or scaffolds (Figure 3). The largest number of genes (10 genes) was located on chr6, while chr2 contained the fewest genes (2 genes). A pair of tandem duplications (Csa_4G043830 and Csa_4G043840) belonging to the HAM subfamily was located on chr4 (marked with green in Figure 3). Ka/Ks = 0.2068 for this pair of genes (<1), indicating purification and selection during the process of evolution. Intra-species analysis results by MCscanX software substantiated this pair of tandem duplications, and the six pairs of segmental duplications were shown in Figure 4; we manually marked collinear genes with different color blocks in Figure 3. We can find most of the genes are located in chr6, which may be an amplification by segmental duplication.

### 2.5. Functional Annotations of GRAS Genes in Cucumber

**Figure 5:**
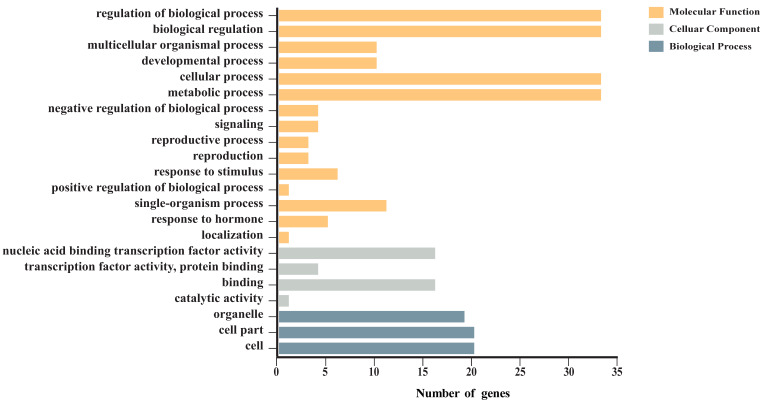
GO enrichment of CsaGRAS transcription factors in cucumber. According to the secondary terminology, the annotation results are divided into three ontology categories and distinguished by different colors.

We analyzed enrichment of GO (gene ontology) and KEGG (Kyoto Encyclopedia of Genes and Genomes) terms associated with the *CsaGRAS* genes to explore their functions. According to GO enrichment analysis, the 37 GRAS family members were divided into three ontology categories: biological process, cellular component and molecular function (Table S4). These GO terms were then divided into 22 functional terms (Figure 5; Supplementary Table S5). We predicted that the GRAS family genes in cucumber are involved in many processes of plant regulation, with 33 genes involved in biological regulation, cellular processes and metabolic processes; 16 genes showed potential transcription factor activity and binding functions.

### 2.6. Interaction Network Analysis of GRAS Genes in Cucumber

**Figure 6:**
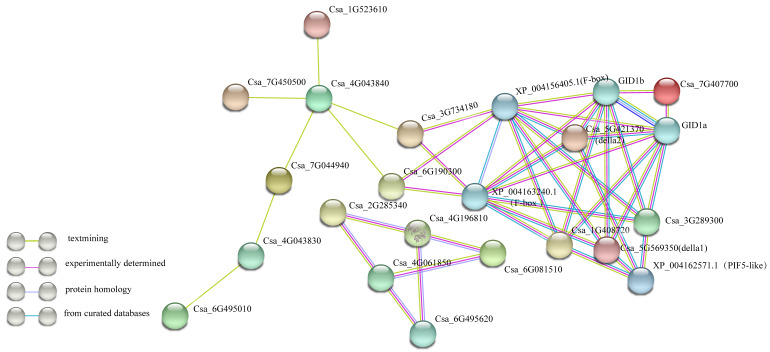
Interaction network among CsaGRAS families in cucumber. Specific protein interactions between GRAS transcription factors in cucumber were determined using String (https://string-db.org/) [43]. The colorful lines represent the confirmed way of conclusion.

To explore the relationships among cucumber GRAS transcription factors during their regulatory functions, we constructed an interaction network (Figure 6). Of the 37 proteins belonging to the GRAS family, four contained transcriptional regulator DELLA protein N-terminal domains (Csa_3G289300, Csa_5G421370, Csa_1G408720, Csa_5G569350). To explore the relationships between CsaGRAS family transcription factors in more detail, we added five genes (XP_004156405.1,*GID2*-like; XP_004163240.1,*GID2*-like protein;XP_004148878.1, *GID1b*; XP_004135640.1; *GID1a*; XP_004162571.1, *PIF5*-like transcription factor) that would interact with the DELLA proteins and relieve the inhibition of gibberellin signal transduction (GA signaling negative regulation pathway) (Table S6). Our gene network did not include 18 unassociated GRAS family genes. We presumed that as the core gene in GRAS family genes correlation, Csa_4G043840 can interact with all five family genes, so that it may respond to a variety of plant processes, such as responsive grafting [41] and soybean mosaic virus [42]. These interrelationships will provide a reference for studying the regulatory functions of GRAS genes in cucumber.

### 2.7. Temporary and Spatial Expression Analysis of CsaGRAS Genes Under Chilling Stress

**Figure 7:**
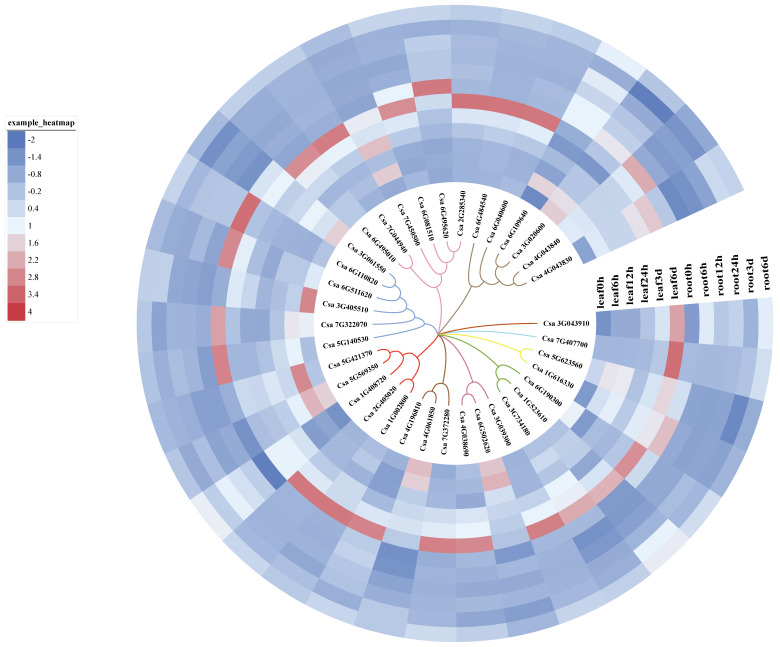
Expression profile of *CsaGRAS* genes in different tissues under cold treatment duration. The data are clustered according to phylogenetic tree.

We used quantitative reverse-transcription (RT)-PCR and heat maps to analyze the effect of cold stress on GRAS gene expression in the leaves and roots of cucumber seedlings. The 37 GRAS genes responded to cold, with expression of some genes such as Csa5G421370, Csa7G322070, Csa7G450500 and Csa6G109640 induced under short-term cold conditions in leaves (Figure 7; Supplementary Table S7). Some *GRAS* genes showed differences in expression in the leaves and roots with increasing duration of cold treatment, such as Csa1G408720, Csa1G523610, Csa3G04391 and Csa3G061550. On the contrary, the expression levels of some genes (such as Csa6G495620 and Csa6G081510) in leaves and roots were reversed (increase in leaves, decrease in roots) with increased duration of cold treatment. GO annotations revealed that these two genes are SHR-like proteins (Table S8), which regulate leaf development. Higher gene expression was observed at 6 h and 6 days of cold treatment in leaves and at 0 h of cold treatment in roots, suggesting that GRAS genes are associated with cold tolerance in leaves (Figure 7).

## 3. Discussion

The GRAS gene family has been analyzed in many plants, such as tea (*Camellia sinensis*) [24] and tomato (*Solanum lycopersicum*) [23], laying an important foundation for functional analysis of GRAS family members. However, the characteristics and functions of GRAS family members in cucumber are unclear. We therefore used bioinformatics to analyze their structure and function. We identified 37 GRAS genes in cucumber based on existing information about GRAS family proteins and a domain search in the gff3 of cucumber. Taking advantage of this functional information from other species, we constructed a phylogenetic tree of GRAS proteins and used it to explore the evolutionary relationships of the CsaGRAS family and identify genes potentially involved in responses to cold stress. Structural analysis showed that the arrangement and number of protein motifs in each CsaGRAS subfamily were different, but similarity was high within each subfamily. Differences in conserved regions of 10 motifs suggested different functions for each subfamily, with specific mutation of non-conserved amino acids contributing to functional separation [44] during evolution. Duplication is considered the main cause of gene family expansion [45,46]. We screened a pair of tandem duplications in the HAM subfamily and six pairs of collinear genes using the BLAST method and MCScanX software (Plant Genome Mapping Laboratory, University of Georgia, Georgia, USA), respectively. Ka/Ks values suggested they had undergone purification and selection to maintain their important biological functions. To some extent, tandem duplications and segmental duplications were important factors leading to the uneven distribution of expansive gene families on chromosomes [47].

Sequence characteristics of *CsaGRAS* genes were more similar to those of *Arabidopsis* than those of tomato. High sequence similarity often indicates a similar function in different species [48]. Therefore, we mainly referred to *Arabidopsis* for GO annotation and interaction network analysis of GRAS proteins. Consistent with previous findings, we observed that *CsaGRAS* genes are likely involved in a variety of biological, developmental, multicellular organismal and metabolic plant processes. The DELLA subfamily can interact with GID1 to participate in GA signal transduction. The CBF/DREB1 pathway is important in the cold signal response of *Arabidopsis* [32,33], in which *CBF1* reduces the level of bioactive gibberellin. This process modulates the accumulation of DELLA proteins, enhancing cold tolerance [34], so that some *CsaGRAS* transcription factors of the DELLA subfamily may be downstream factors of the CBF pathway for cold tolerance. The CBF pathway is not only induced by cold, but also regulated by light at room temperature. Phytochrome interacting factors (PIF3, PIF4 and PIF7), which can negatively regulate the phyB signal pathway, can directly bind to the *CBF* promoter and negatively regulate the expression of *CBF* [49,50,51]. Moreover, some transcription factors have been substantiated that not only positively regulate the phytochrome signaling pathway but also have transcriptional activity. So, we speculated that some transcription factors of cucumber (Csa_3G289300, Csa_1G408720, Csa_4G061850) may also be involved in the cold regulation pathway of CBF. We constructed a larger interaction network on the string website and found that the guessed genes did interact with the PIF5-like transcription factor.

Transcription factors of the transportable SHR subfamily can interact with those of the SCR subfamily to regulate leaf development [13,29,30]. Our heat map of the responses of GRAS genes to cold indicated that most of the GRAS family genes could respond to cold and were highly responsive in leaves. We believe that GRAS family genes play an important role in the cold tolerance of cucumber, mainly functioning in leaves; however, the response site is not necessarily the leaf. There are early and late cold-responsive genes in almost every subfamily, suggesting some genes may be able to respond to cold-induced stress. Cold induction is often beneficial, for example, transient cold-induced male meiotic recovery [52]. Therefore, we hypothesize that each subfamily plays a different role in responding to cold stress. In particular, the SHR and SCR subfamilies may regulate shoot cold tolerance in response to root cold-tolerance signals. GA can participate in a variety of regulatory processes as a signal whose transduction requires a GA–GID1–DELLA complex. It is therefore of great interest to study the role of cucumber GRAS family genes in cold tolerance by associating the phenotype induced by cold with GA signal transduction.

Gene structure, motif logo, chromosome distribution and evolution analysis show that the CsaGRAS gene family has highly conserved domains and complex evolutionary relationships. The expression patterns combined with functional annotations and interaction network provided the candidate genes for cold tolerance and a new perspective for understanding the molecular evolution and biological function of the GRAS gene family.

## 4. Materials and Methods

### 4.1. Screening and Identification of GRAS Genes in Cucumber

To establish a local database, the *Cucumis sativus* genome (cds, pep, dna and gff3) was downloaded from Ensembl (http://plants.ensembl.org/index.html) and GRAS.hmm (PF03514) was downloaded from Pfam (http://pfam.xfam.org/family/PF03514#curationBlock). The gff3 file was screened twice for GRAS proteins based on GRAS.hmm using HMMER 3.1 software (National Institutes of Health, US, grant number R01HG009116) (E-value < 1.2e ^−28^). All GRAS protein sequences obtained were combined with search results from SMART (http://smart.embl.de/), NCBI (https://www.ncbi.nlm.nih.gov/cdd/) and Pfam (http://pfam.xfam.org/) databases. Proteins lacking complete GRAS domains were identified by manual examination. GRAS proteins were identified using ExPASy (http://web.expasy.org/protparam/) [53].

### 4.2. Phylogenetic Analysis of GRAS Proteins in Cucumber

*Arabidopsis* and tomato are commonly used model plants in vegetable research. Therefore, multiple sequence alignment and reconstruction of an evolutionary tree of the GRAS families of cucumber, *Arabidopsis* and tomato were performed using MEGA-X software (Center of Evolutionary Functional Genomics Biodesign Institute, Arizona State University, Tempe, AZ, USA) [54], with full-length GRAS protein sequences. GRAS sequences from *Arabidopsis* were downloaded from TAIR (http://www.arabidopsis.org/), and those for tomato were obtained from the research of Niu et al. [23]. Evolutionary trees were divided into 10 groups according to the classification of GRAS proteins in *Arabidopsis* and tomato [3,23].

### 4.3. Structural Analysis of GRAS Genes in Cucumber

Location of exons, coding sequences and untranslated regions on cucumber chromosomes were extracted from the gff3 file to draw a gene structure map. MEME (http://meme-suite.org/tools/meme) [55] was used to identify potentially conserved motifs in 37 complete CsaGRAS amino acid sequences with default parameters, except motif number was set to 10. Information extraction, preliminary drawing of the exon-intron structure and motif analysis were completed using TBtools (HuaZhong Agricultural University, Wuhan, China) [56]. The positions of genes on chromosomes and chromosome lengths were extracted from gff3 and dna files, respectively, and a chromosome location map was drawn using Mapchart 2.32 (Wageningen University &Research, Wageningen, The Netherlands) (https://www.wur.nl/en/Research-Results/Research-Institutes/plant-research/biometris/Software-Service/Download-MapChart.htm).

### 4.4. Evolution Analysis of GRAS Gene in Cucumber

Tandem duplication of *CsaGRAS* was identified using the BLAST method, and Ka/Ks values were calculated using KaKs_Calculator2.0 software (CAS Key Laboratory of Genome Sciences and Information, Beijing Institute of Genomics, Chinese Academy of Sciences, Beijing, China) [57,58,59]. Tandem duplication was defined as a gene pair whose alignment value is more than 70% between the alignment sequence and the longest sequence with the genes residing within 100 kb [60]. Then, we used MCScanX software (Plant Genome Mapping Laboratory, University of Georgia, Athens, GA, USA, http://chibba.pgml.uga.edu/mcscan2/) [61] to analyze syntenic regions (segmental duplications) to verify and supplement the blast results. The collinear relationship of *CsaGRAS* genes was visualized by Circos-0.69-6 software (Canada’s Michael Smith Genome Sciences Centre, Vancouver, BC, Canada) [62].

### 4.5. Gene Ontology Enrichment and Interaction Network Analysis

GO enrichment analysis was performed using the OmicShare tools, a free online platform for data analysis (http://www.omicshare.com/tools). Specific protein interactions between GRAS transcription factors in cucumber were determined using String (https://string-db.org/) [43].

### 4.6. Plant Materials, Growth Conditions and Cold Treatment

Cucucmber (*Cucumis sativus* L. ‘Xintaimici’, laboratory homozygous material) was used in this study. One hundred seeds were soaked in warm water, placed in aseptic water at 55 °C for 20 min, then transferred to room temperature water at 28 °C for 4 h before germinating in a dark chamber at 28 °C for 1 day. Germinated seeds were sown in a 50 hole seedling plate with substrate (peat: vermiculite: perlite, volume ratio 2:1:1). After sowing, seeds were placed in a light incubator for growth (relative humidity: 70%, 16 h/8 h light/dark; 28 °C/18 °C day/night; light intensity 190–600 μmol m^−2^ s^−1^). When plants reached the second true leaf stage, they were treated at 4 °C.

Leaves and roots of two-leaf cucumber seedlings were sampled at 0 h, 6 h, 12 h, 24 h, 3 days and 6 days after cold treatment (relative humidity: 70%; 16 h/8 h light/dark; 4 °C/4 °C day/night; light intensity: 190–600 μmol m^−2^ s^−1^). Three independent biological replicates were performed, each comprising 3–6 individual plants.

### 4.7. RNA Isolation and cDNA Synthesis

Total RNA was extracted from different cucumber tissues using RNA plant Plus Reagent (Huayueyang Biotech, Co., Beijing, China) according to the manufacturer’s instructions. To determine RNA quality and concentration, 1 µl of each RNA sample was subjected to agarose gel electrophoresis (2%, agarose, 1× TBE gel) and quantified using a NanoDrop 2000 (Thermo Fisher Scientific, Waltham, MA, USA). cDNA was synthesized according to the instructions of the PrimeScript^TM^ RT reagent kit with gDNA Eraser (Perfect Real Time, Takara Biomedical Technology Co., Ltd. (Beijing, China) ).

### 4.8. Quantitative RT-PCR Assays

Quantitative RT-PCR was performed using TB Green^TM^ Premix Ex Taq^TM^ (Tli RNaseH Plus) (Cat#RR420A, Takara Biomedical Technology Co., Beijing, China) on a QuantStudio^TM^ 6 Flex real-time PCR system (Applied Biosystems, Foster City, CA, USA), following the manufacturers’ instructions. At least 3–5 technical replicates were performed. Stage 1: 1 cycle at 50 °C for 2 min; Stage 2: 1 cycle at 95 °C for 10 min; Stage 3: 40 cycles at 95 °C for 15 s, 60 °C for 1 min; Dissociation stage: 15 s at 95 °C, 15 s at 60 °C, 15 s at 95 °C. Primers used are listed in Table S9. Values were normalized by comparing them with those of *Actin* (*CsaACTN7*, Csa6G484600) using the ∆∆Ct method, and standardized using the z-score (zero-mean normalization) method. The original Ct value of *CsaACTIN7* in Table S10 indicated that the gene expression was stable in cold treatment. A heat map was drawn using iTOL (https://itol.embl.de/) to visualize the RT-PCR results.

## Figures and Tables

**Table 1:** Comparison of GRAS transcription factors among different species.

Species	Classification										Total
	PAT1	SHR	HAM	DELLA	LISCL	SCR	LAS	SCL3	Gv6	SCL28	
*Solanum Lycopersicum*	11 (0.215686)	5 (0.098039)	7 (0.137255)	5 (0.098039)	7 (0.137255)	3 (0.058824)	2 (0.039216)	2 (0.039216)	7 (0.137255)	2 (0.039216)	51
*Cucumis sativus* L.	6 (0.162162)	6 (0.162162)	6 (0.162162)	6 (0.162162)	3 (0.081081)	3 (0.081081)	3 (0.081081)	2 (0.054054)	1 (0.027027)	1 (0.027027)	37
*Arabiopsis Thaliana*	6 (0.176471)	4 (0.117647)	5 (0.147059)	5 (0.147059)	7 (0.205882)	2 (0.058824)	3 (0.088235)	1 (0.029412)	0 (0.0000)	1 (0.029412)	34
**total**	23	15	18	16	17	8	8	5	8	4	122

## Supplementary Materials

The following are available online at https://www.mdpi.com/1422-0067/21/11/3857/s1.
